# Personalized Dynamic Prediction Model for Biopsy Timing in Patients With Prostate Cancer During Active Surveillance

**DOI:** 10.1001/jamanetworkopen.2024.54366

**Published:** 2025-01-16

**Authors:** Ivo I. de Vos, Daan Nieboer, Mark Frydenberg, Christian P. Pavlovich, Mieke van Hemelrijck, Lui Shiong Lee, Antti Rannikko, Anders Bjartell, Axel Semjonow, Ewout W. Steyerberg, Monique J. Roobol

**Affiliations:** 1 Erasmus MC Cancer Institute, University Medical Center Rotterdam, Rotterdam, the Netherlands; 2 Cabrini Institute, Cabrini Health, Monash University, Sydney, Australia; 3 The James Buchanan Brady Urological Institute, Johns Hopkins University, Baltimore, Maryland; 4 King’s College London, London, United Kingdom; 5 Guy’s and St Thomas’ NHS Foundation Trust, London, United Kingdom; 6 Sengkang General Hospital, Singapore; 7 Helsinki University Hospital, Helsinki, Finland; 8 Department of Urology and Research Program in Systems Oncology, University of Helsinki, Helsinki, Finland; 9 Skåne University Hospital, Malmö, Sweden; 10 Prostate Center, University Hospital Muenster, Muenster, Germany; 11 Department of Biomedical Data Sciences, Leiden University Medical Center, Leiden, the Netherlands

## Abstract

**Importance:**

Active surveillance (AS) for patients with prostate cancer (PC) often includes fixed repeat prostate biopsies that do not account for the varying risk of reclassification to significant disease. Given the invasive nature and potential complications of biopsies, a personalized approach is needed to balance the burden of biopsies with the risk of missing disease progression.

**Objective:**

To develop and externally validate a dynamic model that predicts an individual’s risk of PC reclassification during AS.

**Design, Setting, and Participants:**

This prognostic study developed a dynamic prediction model using data from the Prostate Cancer Research International: Active Surveillance (PRIAS) study, which was initiated in 2006. Follow-up was truncated until April 2023. External validation was conducted using cohorts from the world’s largest centralized AS database, the Global Action Plan Prostate Cancer Active Surveillance initiative database. The PRIAS study is a multicenter, prospective, web-based cohort study monitoring patients undergoing AS, involving more than 175 academic, nonacademic, and private centers across 23 countries worldwide. For the development and external validation of the model, all patients diagnosed with Grade Group 1 PC who underwent at least 1 baseline or follow-up magnetic resonance imaging (MRI) and 1 follow-up biopsy were included. Data were analyzed from September 2023 to January 2024.

**Exposures:**

AS, including prostate-specific antigen (PSA) tests, MRI, and prostate biopsies according to a fixed follow-up schedule.

**Main Outcomes and Measures:**

A joint model for longitudinal and time-to-event data was used to predict reclassification to Grade Group 2 or greater on repeat biopsy using predefined baseline and repeated clinical characteristics. Performance was assessed using time-dependent area under the receiver operating characteristic curve and negative predictive value.

**Results:**

The development cohort included 2512 patients (median [IQR] age, 65 [59-69] years). Characteristics significantly associated with a higher risk of reclassification were increased age, higher PSA and velocity, lower prostate volume, a suspicious lesion on MRI, and no previous negative biopsy findings. Depending on the threshold and time point used, the model demonstrated a negative predictive value of 86% to 97%. External validation included 3199 patients from 9 other cohorts. The time-dependent area under the curve ranged from 0.81 to 0.84 in the development cohort and 0.52 to 0.90 at external validation.

**Conclusions and Relevance:**

In this prognostic study, the developed dynamic risk model effectively identified patients at low risk of PC reclassification during AS. After prospective validation, this model may support personalized, risk-based AS and reduce the burden of unnecessary biopsies.

## Key Points

**Question:**

Can a dynamic model accurately predict risk of prostate cancer (PC) reclassification during active surveillance (AS)?

**Findings:**

In this prognostic study, a model was developed using data from 2512 prospectively followed patients in AS and externally validated with 3199 patients from 9 other cohorts. The model demonstrated a high negative predictive value (86%-97%) and time-dependent area under the receiver operating characteristic curve (development cohort: 0.81-0.84; external validation: 0.52-0.90), thereby effectively identifying patients at low risk of reclassification to significant PC.

**Meaning:**

These findings suggest that the dynamic risk model enables personalized, risk-based AS of patients with low-risk PC, which could reduce unnecessary biopsies and associated patient burden.

## Teaser

This prognostic study develops and externally validates a dynamic model to predict risk of prostate cancer Grade Group reclassification among patients undergoing active surveillance.

## Introduction

Over the past decade, active surveillance (AS) has increasingly become recognized as the standard of care for patients with Grade Group (GG) 1 prostate cancer (PC).^1,2^ AS aims to avoid unnecessary radical treatment by monitoring patients using a combination of prostate-specific antigen (PSA) testing, digital rectal examinations (DRE), imaging, and prostate biopsies. When there is evidence of tumor progression, the patient is offered radical treatment, preserving the opportunity for curative intervention. While this approach can reduce overtreatment and its treatment-related adverse effects, timely prostate biopsies are crucial for confirming reclassification without significant delay. However, these biopsies are invasive, uncomfortable, and not without risk of infection or significant bleeding.^3^ Consequently, patients may be reluctant to undergo repeat biopsies, leading to poor adherence, which has been linked to worse oncologic outcomes.^4,5,6^ In fact, some patients may choose to abandon AS and opt for treatment due to the physical and psychological burden caused by prostate biopsies.^7^ Moreover, despite substantial cost savings over immediate treatment, prostate biopsy remains the most expensive element of AS.^8,9^ Considering the above and the fact that 66% to 87% of biopsies within a fixed AS algorithm can be considered redundant because they do not show histological progression,^10,11,12^ it is clear that AS algorithms need to be improved.

AS is usually conducted with a one-size-fits-all approach,^13^ with the European Association of Urology (EAU) guidelines recommending to perform repeat biopsies every 2 to 3 years to minimize delay in detection of cancer progression.^14^ However, this fixed approach does not account for the significant variation in cancer progression rates among patients undergoing AS. Patients with fast-progressing cancers may benefit from frequent biopsies, while patients with slow-progressing or nonprogressing cancers may undergo unnecessary, burdensome procedures. Personalizing the decision to conduct biopsies based on an individual’s risk may aid in balancing the burden of biopsies against the risk in detecting reclassification. Several models have previously been developed to predict the presence of higher-grade disease in patients undergoing AS.^15,16,17,18^ However, these models either rely solely on clinical characteristics at time of diagnosis or do not take into account magnetic resonance imaging (MRI) findings. Therefore, this study aimed to develop and validate a dynamic model that predicts an individual’s risk of reclassification during AS based on baseline characteristics and repeated clinical measurements, including age at diagnosis, PSA level, prostate volume, MRI findings, and previous biopsy results.

## Methods

This prognostic study was approved by international steering committee of the Prostate Cancer Research International: Active Surveillance (PRIAS) study, which was approved by the medical ethical committee of the Erasmus University Medical Centre and, dependent of local regulations, local committees. All participants provided written informed consent. This study is reported following the Transparent Reporting of a Multivariable Prediction Model for Individual Prognosis or Diagnosis (TRIPOD) reporting guideline.

Patients from the PRIAS study were included in this analysis as the development cohort. PRIAS is a multicenter, prospective cohort study that was initiated in 2006 and maintains a web-based register,^19^ allowing clinicians to submit data of patients with PC who opted for AS after diagnosis. The study involves more than 175 academic, nonacademic, and private practices across 23 countries worldwide, representing daily clinical practice worldwide. Based on the a patient’s individual data, the PRIAS website generates recommendations on how to continue AS according to the fixed follow-up protocol.

The initial inclusion criteria for the PRIAS study at time of diagnosis were GG 1, clinical stage T2c or lower, PSA 10 ng/mL or less (to convert to micrograms per liter, multiply by 1), 2 or fewer core results positive for PC, PSA-density 0.2 ng/mL/cm^3^ or less, and the patient was fit for curative treatment.^20^ In 2015, the introduction of MRI in PC diagnosis led to the elimination of restrictions on the number of total positive cores for GG 1 disease. In addition, the inclusion criteria were expanded in 2020 to accommodate a higher PSA (≤20 ng/mL) and PSA density (≤0.25 ng/mL/cm^3^) if MRI was used at inclusion. The follow-up schedule includes PSA measurements, DRE, MRI, and repeat biopsies on a fixed schedule (eTable 1 in Supplement 1). Repeat biopsies are recommended at 1, 4, 7, and 10 years after inclusion, and until 2015, yearly biopsies were advised when PSA doubling time was less than 10 years. After 2015, yearly biopsies were only recommended if the MRI showed progression.

### Inclusion Criteria for Model Development and Validation

For the development and external validation of the model, we included all patients diagnosed with GG 1 disease who underwent at least 1 baseline or follow-up MRI and 1 follow-up biopsy (ie, after the initial diagnostic biopsy). Follow-up was truncated until April 2023. The developed model was externally validated on cohorts within the Global Action Plan Prostate Cancer Active Surveillance initiative (GAP3) database. GAP3 was initiated in 2018 with the aim of creating a centralized AS database by combining patient data from established AS cohorts worldwide. The primary objective of GAP3 is to reach a global consensus with uniform guidelines on the selection and monitoring of patients with low-risk PC.^21^ Currently, the GAP3 database includes data of 26 999 patients undergoing AS from 25 cohorts in 15 countries (version 4.1.1). We included centers with more than 100 eligible patients. The cohorts meeting this criterion were from the University of California, San Francisco; Johns Hopkins University; Memorial Sloan Kettering Cancer Centre; University College London; University of Cambridge; Michigan Urological Surgery Improvement Collaborative; National Cancer Institute of Milan; Oncological Institute of Valencia; and University of Technology Sydney.

### Statistical Analysis

We developed a dynamic model predicting the risk of reclassification to GG 2 or greater on repeat biopsy using a statistical model that combines analyses of longitudinal and time-to-event data. This joint model incorporated predefined established risk factors based on prior research,^22,23^ including age at diagnosis, PSA, prostate volume, number of previous benign findings on biopsy, and whether a suspicious lesion (Prostate Imaging Reporting & Data System or Likert ≥3) was present on MRI. Multiple generalized linear mixed models were used for longitudinal analyses of log-transformed PSA, log-transformed prostate volume, the presence of a suspicious lesion, and for having a biopsy without cancer present. This model was joint with a relative risk submodel for prediction of the time to reclassification. Patients without detected reclassification were censored at their last biopsy.

The correlations between repeated measurements of the different included risk factors were modeled using patient-specific random effects. Using the patient-specific random effect, we estimated patient-specific curves of the evolution of PSA results, prostate volume, and the likelihood of a suspicious lesion. Subsequently, we calculated the mathematical derivative of a patient’s fitted PSA profile to estimate the patient’s instantaneous PSA velocity. The instantaneous PSA velocity, a stronger predictor than commonly used mean PSA velocities,^24^ was calculated by estimating the best linear unbiased predictor of the patient-specific profile of PSA over time, taking into account all previous measurements, and was the mathematical derivative of the patient-specific profile at the point at which a prediction of reclassification was made (eFigure 1 in Supplement 1).

In the relative risk submodel, we included age at diagnosis, fitted log2-PSA, instantaneous log2–PSA velocity, fitted log2–prostate volume, fitted value for having benign biopsy findings, and fitted value for suspicious lesion.

All statistical analyses were conducted using R statistical software version 4.3.1 (R Project for Statistical Computing). The submodels were estimated jointly using the R package JMbayes. The statistical formula of the model is provided in eMethods in Supplement 1. *P* values were 2-sided, and statistical significance was set at *P* ≤ .05. Data were analyzed from September 2023 to January 2024.

#### Performance Assessment

Prognostic discrimination of the model was evaluated with the time-dependent area under the receiver operating characteristic curve (AUC), with the time ranging from 1.5 to 5 years. The starting point of 1.5 years was selected for the time-dependent AUC to include results of confirmatory biopsies occurring just after the 1-year mark. In addition, diagnostic accuracy was evaluated with sensitivity, specificity, negative predictive value (NPV), and positive predictive value across various thresholds. Clinical utility of the developed model was further assessed by the net number of biopsies avoided as derived in a decision curve analysis.^25^ At external validation, we assessed the model’s discrimination (measured by time-dependent AUC) and calibration by comparing the overall risk of upgrading with the mean upgrading risk.

#### Sensitivity Analyses

In sensitivity analyses, we assessed the impact of including only patients with a baseline MRI, as this may better reflect contemporary clinical practice in which patients receive an MRI before diagnosis. In addition, we also examined the model’s performance with GG 3 or greater as the outcome.

## Results

**Figure 1.**
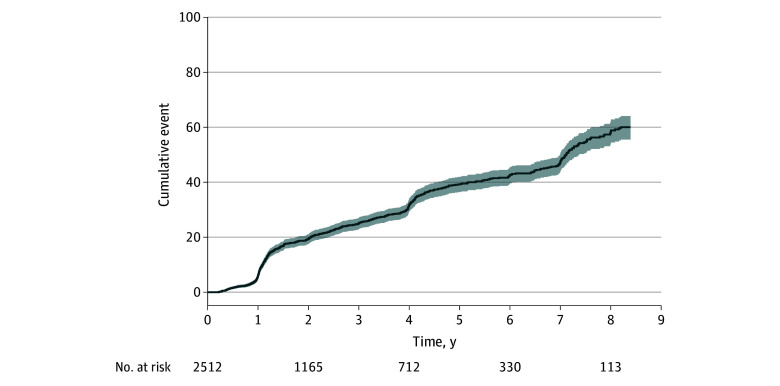
Cumulative Incidence of Reclassification to Grade Group 2 or Greater on Biopsy in the Prostate Cancer Research International: Active Surveillance Development Cohort (n = 2512) Line indicates cumulative incidence; shading, 95% CI.

A total of 2512 patients (median [IQR] age, 65 [59-69] years; median [IQR] PSA, 5.7 [4.6-7.4] ng/mL at time of diagnosis) (Table 1) in the PRIAS database fit the inclusion criteria and were included in the development cohort. During the follow-up period, reclassification to GG 2 or greater was detected in 720 patients (Figure 1). Of these, 194 patients (27%) were reclassified to GG 3 or greater. The median (IQR) available follow-up time of patients in the development cohort who did not experience upgrading was 1.6 (1.1-4.1) years. After 5 years, 400 patients were still at risk (Table 1).

**Table 1.** Patient Characteristics of the Development and External Validation Cohorts

Characteristic	Median (IQR)									
	PRIAS (n = 2512)	UCSF (n = 485)	JHU (n = 952)	MSKCC (n = 256)	UCL (n = 216)	Cambridge (n = 106)	Milan (n = 488)	MUSIC (n = 295)	Valencia (n = 195)	Sydney (n = 206)
Age at diagnosis, y	65 (59-69)	62 (58-67)	65 (61-69)	61 (56-64)	63 (57-67)	64 (59-68)	64 (58-69)	64 (59-68)	64 (60-69)	60 (55-65)
Year of diagnosis	2017 (2014-2019)	2014 (2012-2016)	2013 (2009-2017)	2009 (2008-2010)	2011 (2009-2013)	2012 (2011-2014)	2016 (2013-2018)	2016 (2015-2017)	2013 (2012-2014)	2011 (2009-2013)
PSA at diagnosis, ng/mL	5.7 (4.6-7.4)	5.9 (4.4-8.1)	5.0 (4.0-6.6)	4.5 (3.2-6.0)	5.8 (4.5-8.0)	6.5 (5.1-8.5)	5.7 (4.4-7.5)	5.2 (4.3-6.8)	5.0 (3.6-7.1)	4.9 (3.4-6.7)
Prostate volume at diagnosis, mL	46 (37-60)	42 (31-58)	47 (36-64)	49 (40-60)	45 (33-61)	48 (38-64)	47 (35-66)	43 (33-57)	40 (30-52)	41 (30-56)
PSA-density at diagnosis, ng/mL^2^	0.13 (0.09-0.16)	0.14 (0.10-0.19)	0.10 (0.07-0.14)	0.09 (0.06-0.13)	0.13 (0.09-0.19)	0.13 (0.10-0.21)	0.12 (0.08-0.16)	0.13 (0.09-0.18)	0.12 (0.09-0.18)	0.11 (0.08-0.16)
Patients with baseline MRI, No. (%)	852 (34)	434 (89)	300 (32)	0	216 (100)	60 (57)	247 (51)	53 (18)	113 (58)	206 (100)
Patients with suspicious lesion in baseline MRI, No. (%)	473 (56)	259 (59)	242 (71)	NA	115 (53)	51 (85)	147 (59)	50 (94)	49 (42)	98 (48)
No. of MRI taken, No. (%)										
1	1057 (42)	216 (45)	412 (43)	113 (44)	24 (11)	52 (49)	216 (44)	236 (80)	104 (52)	206 (100)
2	805 (32)	167 (34)	295 (31)	89 (35)	46 (21)	30 (28)	148 (30)	46 (16)	51 (26)	0
≥3	652 (26)	102 (21)	245 (26)	54 (21)	146 (68)	24 (22)	124 (25)	13 (4.4)	40 (21)	0
No. of biopsies taken, No. (%)										
1	1197 (48)	133 (27)	343 (36)	23 (9.0)	174 (81)	59 (56)	206 (42)	255 (86)	89 (45)	108 (52)
≥2	1315 (52)	352 (73)	609 (64)	233 (91)	42 (19)	47 (44)	282 (58)	40 (14)	106 (55)	98 (48)
Negative biopsy results during follow-up	1145 (46)	69 (14)	476 (50)	183 (72)	38 (18)	40 (38)	211 (43)	-	101 (51)	63 (31)
Actuarial risk of GG reclassification on biopsy, %										
2	39	45	26	7	41	41	31	73	29	37
≥3	12	5	2	0	0	5	5	21	2	4
Follow-up time without reclassification										
Time, y	1.7 (1.1-4.1)	0.70 (0-4.4)	3.88 (2.0-6.1)	6.50 (3.6-9.2)	3.12 (0.99-4.6)	0.83 (0.42-2.3)	2.92 (1.2-4.7)	1.18 (0.76-2.2)	3.94 (2.2-6.0)	4.65 (3.1-7.2)
No. at risk at 3 y	950	191	14	78	585	145	276	226	53	146
No. at risk at 5 y	400	105	8	37	333	70	184	188	6	82

Abbreviations: GG, Grade Group; JHU, Johns Hopkins University; MRI, magnetic resonance imaging; MSKCC, Memorial Sloan Kettering Cancer Centre; MUSIC, Michigan Urological Surgery Improvement Collaborative; NA, not applicable; PRIAS, Prostate Cancer Research International: Active Surveillance; PSA, prostate-specific antigen; UCL, University College London; UCSF, University of California, San Francisco.

SI conversion factor: To convert PSA to micrograms per liter, multiple by 1.

### Model Performance in Development Cohort

**Figure 2.**
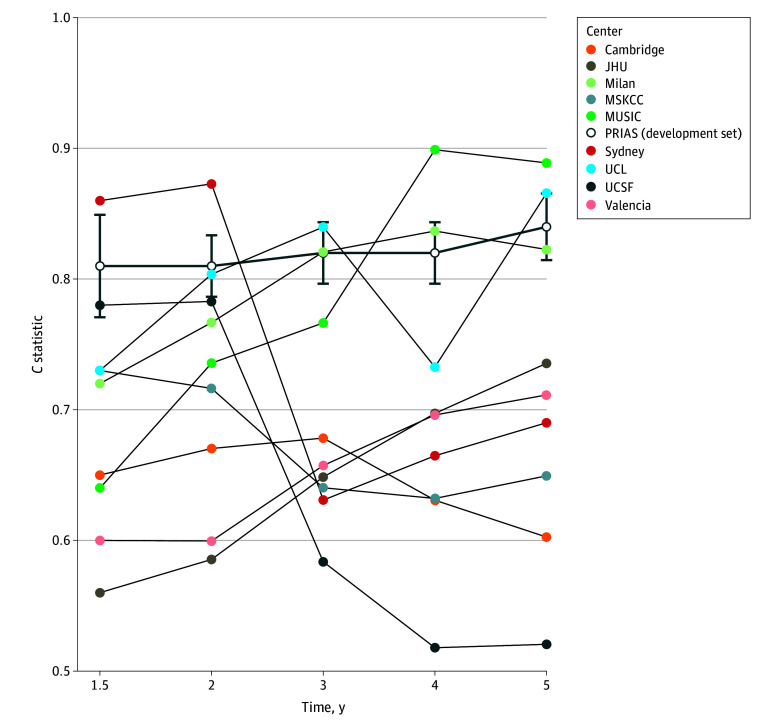
Time-Dependent Area Under the Receiver Operating Characteristic Curve (AUC) at Model Development and External Validation The time-dependent AUC measures the ability of the developed model to discriminate between patients who showed reclassification and no reclassification and were at risk at that time. The bold dark blue line is the apparent time-dependent AUC within the development set Prostate Cancer Research International: Active Surveillance (PRIAS) and includes 95% CIs (whiskers) around the time-dependent AUC. The different colored lines denote the time-dependent AUC within different cohorts at external validation. JHU indicates Johns Hopkins University; MSKCC, Memorial Sloan Kettering Cancer Centre; MUSIC, Michigan Urological Surgery Improvement Collaborative; UCL, University College London; UCSF, University of California, San Francisco.

In the multivariable model, variables significantly associated with a higher risk of reclassification to GG 2 or greater were increased age at diagnosis, higher PSA, higher PSA velocity, and a suspicious lesion on MRI (Table 2). A higher prostate volume and 1 or more previous negative biopsies were associated with a lower risk of reclassification. The time-dependent AUC ranged from 0.81 to 0.84 in the PRIAS cohort (Figure 2). Sensitivity analysis, restricted to patients who underwent MRI prior to diagnosis (852 patients [34% of the whole cohort]), demonstrated similar hazard ratios and time-dependent AUC (eTable 2 and eTable 3 in Supplement 1). Additionally, when the model’s outcome was defined as GG 3 or greater, similar hazard ratios were observed along with an increased time-dependent AUC (eTable 4 and eTable 5 in Supplement 1).

**Table 2.** Associations of Risk Factors in the Survival Part of the Model

Factor	HR (95% CI)
Age at diagnosis, per 10 y	1.47 (1.30-1.80)
PSA, per doubling^a^	1.42 (1.14-1.74)
PSA velocity (75th vs 25th percentile)	1.24 (1.05-1.47)
Prostate volume, per doubling^a^	0.56 (0.44-0.70)
Suspicious lesion on MRI (vs none)	1.45 (1.14-1.74)
No. of previous negative biopsy findings, per 1 negative finding	0.72 (0.67-0.76)

Abbreviations: HR, hazard ratio; MRI, magnetic resonance imaging; PSA, prostate-specific antigen.

^a^ For example, 8 ng/mL vs 4 ng/mL (PSA) or 60 ng/mL vs 30 ng/mL (prostate volume).

### Model Performance at External Validation

A total of 3199 patients from 9 GAP3 AS cohorts were available for external validation (Table 1). The time-dependent AUC ranged from 0.52 to 0.90, depending on the cohort and number of years after diagnosis (Figure 2). Model calibration of the cohorts is presented in eFigure 2 in Supplement 1.

### Clinical Implications

Table 3 presents the model’s diagnostic accuracy in the PRIAS cohort at various thresholds and time points. Results for the external validation cohorts are presented in eTable 6 in Supplement 1. A threshold of 7.5% at 1.5 years allowed the deferral of 354 of 1000 biopsies, with 11 delayed reclassifications. This yielded a sensitivity of 92% (95% CI, 89%-95%) and a NPV of 97% (95% CI, 95%-98%). Alternatively, a threshold of 15% enabled the deferral of 645 biopsies, with 24 delayed reclassifications, resulting in a sensitivity of 69% (95% CI, 64%-74%) and a NPV of 93% (95% CI, 91%-94%). eFigure 3 in Supplement 1 illustrates 2 PRIAS patients with PSA progression but with different results on MRI and a different prostate volume, highlighting the significance of the model’s dynamic capability. eTable 7 in Supplement 1 summarizes the dynamic risk model.

**Table 3.** Diagnostic Accuracy and Net Benefit in the Development Cohort When Applying the Model at Biopsy Time Points

Threshold probability, %	% (95% CI)				No. per 1000 biopsies		Net biopsies avoided, No. per 100 patients
	Sensitivity	Specificity	NPV	PPV	Biopsies delayed	Detected reclassifications delayed	
**At year 1.5**							
7.5	92 (89-95)	40 (38-43)	97 (95-98)	21 (17-26)	354	11	20
10	84 (80-88)	54 (51-56)	95 (94-96)	24 (19-29)	480	24	24
15	69 (64-74)	70 (68-73)	93 (91-94)	29 (24-34)	645	46	34
20	56 (50-61)	81 (79-83)	91 (90-93)	34 (29-40)	758	66	43
**At year 4**							
7.5	94 (89-99)	17 (14-21)	93 (88-98)	18 (15-22)	155	10	1
10	82 (74-90)	33 (29-37)	90 (86-95)	19 (15-23)	305	30	4
15	60 (50-69)	61 (56-65)	88 (85-92)	23 (18-28)	573	66	11
20	36 (26-46)	78 (74-82)	86 (83-89)	24 (17-31)	756	105	19

## Discussion

In this prognostic study using data from the world’s largest multicenter AS study (PRIAS) and world’s largest centralized AS database (GAP3), we developed and validated a dynamic, joint model that predicts an individual’s risk of upgrading during AS. While AS is intended to prevent overtreatment, most repeat prostate biopsies conducted during AS do not show upgrading (82% of the biopsies in the development cohort) and may be considered unnecessary and potentially harmful for the patient. This personalized model can aid decision-making regarding the necessity of repeat prostate biopsies during AS and may therefore safely avoid unnecessary biopsies. By quantifying the risk, the model enables more tailored counseling, allowing clinicians to clearly communicate individualized risks and benefits. This may help patients better understand their own risk, feel more supported in their decisions about repeat biopsies, and ultimately improve adherence.^26^ In the development cohort, the use of a cutoff of 7.5% at 1.5 years after diagnosis would result in the avoidance of 1 in 3 biopsies, with a delay in detection of reclassification in 11 of 1000 biopsies. If a cutoff of 15% is used, almost two-thirds of all biopsies could be avoided, but with a delay in detection of reclassification in 46 of 1000 biopsies.

It is important to acknowledge the assumption that reclassification is not missed entirely; instead, it is delayed. This is due to the fact that the model is dynamic and can continuously be updated with additional patient data during follow-up, which could eventually identify the reclassification at a later time. For instance, if the model predicts a risk below the threshold and no biopsy is conducted, but reclassification is already ongoing, the patient will continue the follow-up schedule. With a new PSA measure after 6 months, the predicted risk may increase above the threshold, resulting in delayed detection of the reclassification. This raises the question whether this delay affects the patient’s window of cure. Fossati et al^27^ conducted a study analyzing the impact of the time between biopsy and radical prostatectomy on biochemical and clinical recurrence in 2653 patients with localized PC. While Fossati et al^27^ observed a nonlinear association after correcting for other clinical characteristics, this finding only had clinical significance in patients with high-risk PC who received treatment more than a year after diagnosis. As a result, Fossati et al^27^ concluded that surgical treatment could be safely postponed for up to 1 year after diagnosis, even for patients with high-risk PC. Other studies have also failed to provide clear evidence of a significant increase in the risk of oncological outcomes after a delay in treatment.^28,29,30^ Since these were all newly diagnosed patients, these results are not directly translatable to patients undergoing AS. However, even with the likelihood that there is probably some delay in detection in all patients undergoing AS who harbor reclassified disease, studies have shown that patients who upgrade during AS typically have less aggressive disease than those with the same grade found at initial biopsy.^31,32^ Therefore, a delay in detection of 6 to 12 months may not necessarily result in worse oncological outcomes.

Various models have been developed to predict the outcome of repeat biopsies in AS.^15,16,17,18^ Nevertheless, most of these models do not use MRI findings, despite the fact that MRI is currently a widely used tool in clinical AS practice and has been suggested as a means of reducing the need for repeat biopsies.^33^ While the Stratified Cancer Surveillance model does incorporate MRI findings, only the baseline MRI results are taken into account and not the MRI data collected during follow-up, as the model is not dynamic.^18^ Our model’s dynamic nature is particularly relevant for patients whose PSA levels, prostate volume, or MRI findings change over time, as this is significantly associated with the risk of being reclassified to a higher GG, given the predictive value these markers.^34,35^ Our model demonstrated a time-dependent AUC ranging between 0.77 and 0.84, depending on the year after diagnosis, suggesting a good discriminative ability.^36^ It is also important to consider that the time-dependent AUC tends to be more conservative than the standard AUC, since it relies only on the data available up until the time of interest.^37^ Compared with the previous model developed with PRIAS data, which did not incorporate MRI findings and prostate volume, our model showed increased discriminative performance (time-dependent AUC of 0.81-0.84 vs 0.62-0.69), indicating the added value of the additional variables.^17^ Additionally, the incorporation of diverse types of centers in the PRIAS cohort, reflecting clinical practice worldwide, improves the generalizability of the model substantially. This is demonstrated by the moderate to good time-dependent AUC observed in the GAP3 validation cohorts. Furthermore, there were notable differences in the AUC among the cohorts at external validation. These differences may be attributed to variations in inclusion criteria and follow-up protocols, particularly the extent to which MRI and repeat biopsies are performed, affecting the rate of detecting upgrading in follow-up. These differences in incidence of upgrading are also observed in the calibration plots. This highlights, as with most prediction models,^38^ the need for recalibrating the model on the targeted cohort before using the suggested cutoff values used for deciding whether to perform a repeat biopsy.

In a personalized, risk-based surveillance, we would recommend using the model at the time points at which the current fixed PRIAS protocol already recommended repeat biopsies (eg, at year 1, 4, 7). Additionally, the model could be used whenever a new MRI is prompted by a PSA increase to decide on the necessity of an early repeat biopsy. Based on our findings, we suggest using the thresholds of 7.5% and 15% in shared decision-making with the patient regarding repeat biopsy.

### Limitations

There are some limitations to our model. First, the model is only applicable to patients enrolled in AS with GG 1 disease at diagnosis and not for those with GG 2 disease who are increasingly eligible for AS. Nonetheless, the primary aim of the model is to decrease unnecessary biopsies, which is particularly the case for patients with low-risk, GG 1 disease. Conversely, in the case of GG 2 disease, a more cautious approach is necessary, due to the higher risk of adverse pathology at radical prostatectomy.^39^ Second, the end point of the model is any reclassification, including GG 2 disease with favorable characteristics that may remain eligible to continue AS. This makes the end point a conservative one. However, sensitivity analysis, defining the outcome as GG 3 or greater, demonstrated similar or improved performance, indicating its ability to predict higher-grade disease. In addition, predicting the presence of any GG 2 disease is still of interest due to the heterogeneity of GG 2 disease, which depends on factors such as the percentage of Gleason pattern 4, presence of cribriform or intraductal growth patterns, and the number of positive cores. In the future, incorporating these factors into the model may become feasible as more data and follow-up information for such patients become available. Furthermore, external validation of the sensitivity analyses conducted in the development cohort using upfront MRI or GG 3 or greater as the model’s outcome was not feasible due to the low number of patients with upfront MRI or reclassification to GG 3 or greater in other cohorts.

## Conclusions

In this prognostic study, we have successfully developed and validated a multivariable, dynamic risk model for predicting GG reclassification on rebiopsy during AS for PC. The model demonstrated a high sensitivity and NPV, highlighting its ability to effectively identify patients with a low probability of reclassification. After prospective validation, this model may enable personalized, risk-based surveillance, aiding shared decision-making and significantly reducing unnecessary biopsies. This way, the burden of AS can be reduced compared with a conventional one-size-fits-all follow-up approach.

## Notes

Supplement 1. **eMethods.** Statistical Formula of the Model **eTable 1.** Time Table of Current PRIAS Protocol Follow-Up Schedule **eTable 2.** Sensitivity Analysis Restricted to Patients With a Baseline MRI **eTable 3.** Time-Dependent AUC Restricted to Patients With a Baseline MRI **eTable 4.** Sensitivity Analysis With the Outcome of the Model Defined as ≥GG3 **eTable 5.** Time-Dependent AUC With the Outcome of the Model Defined as ≥GG3 **eTable 6.** Diagnostic Accuracy and Net Benefit in External Validation Cohorts When Applying the Model at Moments at Which a Biopsy Was Taken **eTable 7.** Proposed Utilization of the Dynamic Risk Model to Enable Personalized, Risk-Based Surveillance **eFigure 1.** Example of Calculation of Instantaneous Change in PSA **eFigure 2.** Calibration Plots Comparing Predicted Versus Observed Rates of Reclassification in Different Cohorts **eFigure 3.** Two Real-Life Examples of Patients From the PRIAS Cohort With PSA Progression, but With Different Results on MRI and a Different Prostate Volume, Highlighting the Significance of the Model’s Dynamic Capability

Supplement 2.
Data Sharing Statement
