# Supplementary material for: Personalized Dynamic Prediction Model for Biopsy Timing in Patients With Prostate Cancer During Active Surveillance
Source: JAMA Netw Open. 2025 Jan 16;8(1):e2454366. doi: 10.1001/jamanetworkopen.2024.54366 (PMC11739991; doi:10.1001/jamanetworkopen.2024.54366)
Supplement: Supplement 1. — eMethods. Statistical Formula of the Model eTable 1. Time Table of Current PRIAS Protocol Follow-Up Schedule eTable 2. Sensitivity Analysis Restricted to Patients With a Baseline MRI eTable 3. Time-Dependent AUC Restricted to Patients With a Baseline MRI eTable 4. Sensitivity Analysis With the Outcome of the Model Defined as ≥GG3 eTable 5. Time-Dependent AUC With the Outcome of the Model Defined as ≥GG3 eTable 6. Diagnostic Accuracy and Net Benefit in External Validation Cohorts When Applying the Model at Moments at Which a Biopsy Was Taken eTable 7. Proposed Utilization of the Dynamic Risk Model to Enable Personalized, Risk-Based Surveillance eFigure 1. Example of Calculation of Instantaneous Change in PSA eFigure 2. Calibration Plots Comparing Predicted Versus Observed Rates of Reclassification in Different Cohorts eFigure 3. Two Real-Life Examples of Patients From the PRIAS Cohort With PSA Progression, but With Different Results on MRI and a Different Prostate Volume, Highlighting the Significance of the Model’s Dynamic Capability [file jamanetwopen-e2454366-s001.pdf]

Supplemental Online Content

de Vos II, Nieboer D, Frydenberg M, et al. Personalized dynamic prediction model for biopsy timing in patients with prostate cancer during active surveillance. *JAMA Netw Open*. 2025;8(1):e2454366. doi:10.1001/jamanetworkopen.2024.54366

eMethods. Statistical Formula of the Model
eTable 1. Time Table of Current PRIAS Protocol Follow-Up Schedule
eTable 2. Sensitivity Analysis Restricted to Patients With a Baseline MRI
eTable 3. Time-Dependent AUC Restricted to Patients With a Baseline MRI
eTable 4. Sensitivity Analysis With the Outcome of the Model Defined as ≥GG3
eTable 5. Time-Dependent AUC With the Outcome of the Model Defined as ≥GG3
eTable 6. Diagnostic Accuracy and Net Benefit in External Validation Cohorts When Applying the Model at Moments at Which a Biopsy Was Taken
eTable 7. Proposed Utilization of the Dynamic Risk Model to Enable Personalized, Risk-Based Surveillance
eFigure 1. Example of Calculation of Instantaneous Change in PSA
eFigure 2. Calibration Plots Comparing Predicted Versus Observed Rates of Reclassification in Different Cohorts
eFigure 3. Two Real-Life Examples of Patients From the PRIAS Cohort With PSA Progression, but With Different Results on MRI and a Different Prostate Volume, Highlighting the Significance of the Model's Dynamic Capability

This supplemental material has been provided by the authors to give readers additional information about their work.

© 2025 de Vos II et al. *JAMA Network Open*.

eMethods 1

The developed model is a joint model for longitudinal and time-to-event outcomes fitted within a Bayesian framework. As such, no simple regression formula is available to estimate the risk of reclassification on biopsy conditional on the full history of longitudinal measurements of a patient. If researchers aim to externally validate the developed model they can contact the authors to provide risk estimates for given patients. Alternatively a first order approximation can be made on the risk of reclassification using the posterior means of the estimated parameters of the developed models. Here we provide a step-by-step guide to make this approximation.

In our analysis we fitted several mixed models that allowed for the estimation of the trajectories of PSA, prostate volume, having a positive lesion and the number of negative biopsies during active surveillance. Since the PSA measurements are taken every 3 months early during active surveillance and every 6 months when men are longer on active surveillance we were able to estimate subject-specific evolution of PSA over time within men on AS. We have done this by using natural splines with 3 degrees of freedom to estimate the evolution of PSA over time. We included random slopes for these spline terms to allow for subject-specific evolution of PSA. For the other longitudinal models measurements were more sparse we included a random intercept term to account for repeated measurements within men, and allow for subject specific average level within these mixed models.

Table: Longitudinal sub-models and structure of these models

	Type of (sub-)model	Covariates included	Random effects
PSA (log base 2 transformed)	Linear mixed model	Time (transformed using natural splines)	Random intercept and slope for time
Prostate Volume (log base 2 transformed)	Linear mixed model	Time (linear term)	Random intercept
Positive lesion	Random effect logistic regression	Time (linear)	Random intercept
Nr. Neg Bx	Random effect poisson regression	Time (linear)	Random intercept

The first step in estimating the probability of reclassification consists of estimating the patient-specific random effects based on the full history of longitudinal measurements $Y(t)$. This consists of optimizing the following function:

$$\underset{b}{\operatorname{argmax}} \operatorname{Likelihood}(Y(t)|b)\phi_{\Sigma}(b)$$

Where b is the vector of random effects, ϕ the probability density function of a multivariate normal distribution with and variance covariance matrix of the random effects Σ. The full likelihood of the longitudinal measurements can be broken down into the different sub-models.

© 2025 de Vos II et al. *JAMA Network Open*.

The likelihood of observing a positive lesion at time t is given by:

$$P(\text{Positive lesion at time t}) = \text{logit}^{-1}(0.08 - 0.01 \times t + b_{lesion})$$

When no negative lesion is observed the likelihood is 1 minus this probability.

The likelihood of observing a prostate volume of PV at timepoint t is given by:

$$\text{Likelihood}(\text{PV}, \text{t}) = \phi_{5{,}56+0.05\times \text{t}+\text{b}_{\text{PV}},0.33}(\log_2(\text{PV}))$$

Where ϕ is the probability density function of a normal distribution with mean $5{,}56 + 0.05 \times \text{t} + \text{b}_{\text{PV}}$ and standard deviation 0.33.

And the likelihood of observing n negative biopsies at timepoint t is equal to:

$$\text{Likelihood}(\text{n}, \text{t}) = \text{f}_{-2.82\pm 0.16\times \text{t}+\text{b}_{\text{nr negative biopsies}}}(\text{n})$$

Where f is the probability density function of the poisson distribution.

For observing a PSA at timepoint t is given by:

$$\text{Likelihood}(\text{PSA}, \text{t}) = \phi_{\mu,0.33}(\log_2(\text{PSA}))$$

Where μ is given by:

$$\mu(\text{t}) = 2.46 + \text{b}_{\text{psa,intercept}} + \left(\beta_{\text{time}} + \text{b}_{\text{psa}}\right)\times \text{ns}(\text{time})$$

And $\beta_{\text{time}} = (0.12, 0.52, 0.68, 0.95)$ and ns denotes the basis function of a natural spline[1] with knots (0, 0.81, 2, 4.22, 17.5).

To obtain the likelihood of the full history all observations are entered in formula's and multiplied with each other. After obtaining the subject specific random effects individualized predictions of the different sub-models can be obtained to estimate hazard of having a reclassification:

$$\begin{aligned} \text{h}_\text{i}(\text{t}|\text{Y}(\text{t}), \text{age}) = {} & \text{h}_0(\text{t}) \\ & \times \exp\left[0.39 \times \frac{\text{age}}{10} + 0.37 \times \text{P}(\text{Positive lesion at t}) + 0.35 \times \log_2(\text{PSA}(\text{t}))\right. \\ & + 1.08\,\frac{\text{d}\log_2(\text{PSA}(\text{t}))}{\text{dt}} - 0.58 \times \log_2\left(\text{Prostate Volume}(\text{t})\right) \\ & \left. - 0.33 \times \text{Nr.Neg}(\text{t})\right] \end{aligned}$$

Where each of the variables denotes the estimated subject-specific version of the relevant covariate.

And $\text{h}_0(\text{t})$ is the baseline hazard function approximated by B-splines[1] of order 4 with knots at the following positions:

1	0.0003
2	0.0003
3	0.0003
4	0.0003
5	1.17
6	2.17
7	2.67
8	3.16
9	3.66
10	4.16
11	4.66
12	5.16

© 2025 de Vos II et al. *JAMA Network Open*.

13	5.65
14	6.15
15	6.65
16	7.15
17	16.1
18	16.1
19	16.1
20	16.1

© 2025 de Vos II et al. *JAMA Network Open*.

And coefficients:

γ_1	-4.09121
γ_2	-3.70134
γ_3	-3.42978
γ_4	-3.50535
γ_5	-3.71145
γ_6	-3.81403
γ_7	-3.74011
γ_8	-3.51898
γ_9	-3.33886
γ_{10}	-3.34539
γ_{11}	-3.39122
γ_{12}	-3.31742
γ_{13}	-3.10487
γ_{14}	-2.77937
γ_{15}	-2.48658
γ_{16}	-2.26522
γ_{17}	-2.05621

Note that the B-splines are implemented within splines package in R and the B-splines at timepoint t can be evaluated using the following function call

splineDesign(knots = knots, t, ord = 4)

The resulting matrix then needs to be multiplied with the vector of gammas to obtain the baseline hazard at timepoint t. Which can subsequently be used to estimate the survival probability at timepoint t using standard formulas from survival analysis.

1. Hastie, T. J. (1992) Generalized additive models. Chapter 7 of Statistical Models in S eds J. M. Chambers and T. J. Hastie, Wadsworth & Brooks/Cole.

© 2025 de Vos II et al. *JAMA Network Open*.

eTable 1: Time table of current PRIAS protocol follow-up schedule

Year	1					2				3		4		5		6		7	
**Month**	*0****	*3*	*6*	*9*	*12*	*15*	*18*	*21*	*24*	*30*	*36*	*42*	*48*	*54*	*60*	*66*	*72*	*78*	*84*
**PSA-test**	X	X	X	X	X	X	X	X	X	X	X	X	X	X	X	X	X	X	X
**DRE**	X		X		X				X		X		X		X		X		X
**Standard Biopsy***	X				X								X						X
**Evaluation**	X		X		X				X		X		X		X		X		X
**MRI + targeted biopsies***	X	X**			X								X						X

** If PSA-doubling time <10 years: An MRI is recommended every year (only in the years no standard biopsy is taken). Additional biopsies are indicated if MRI shows progression, more lesions or growth of currently known lesion(s).*
*** If no MRI was conducted before diagnosis, it is recommended to perform an MRI, with additional targeted biopsies if lesion is visible on MRI, within three months after diagnosis to confirm active surveillance eligibility.*
**** Time of diagnosis*

eTable 2: Sensitivity analysis restricted to patients with a baseline MRI (n=852)

**Predictor**	**Measure**	**HR (95% CI)**
Age at diagnosis	Per decade	1.17 (0.91, 1.49)
PSA	Per doubling	1.09 (0.78, 1.45)
Change in PSA	75th vs 25th percentile	1.32 (1.09, 1.67)
Prostate volume	Per doubling	0.71 (0.50, 1.07)
Positive lesion on MRI	Yes vs. No	1.43 (1.16, 1.82)
No. of previous negative Bx	Per additional negative biopsy	0.66 (0.58, 0.75)

PSA = prostate-specific antigen; MRI = magnetic resonance imaging; Bx = prostate biopsy

eTable 3: Time-dependent AUC restricted to patients with a baseline MRI (n=852)

	**Development**
**Year**	**PRIAS**
1.5	0.76 (0.71, 0.81)
2	0.77 (0.73, 0.82)
3	0.77 (0.72, 0.82)
4	0.75 (0.68, 0.81)
5	0.80 (0.69, 0.91)

© 2025 de Vos II et al. *JAMA Network Open*.

eTable 4: Sensitivity analysis with the outcome of the model defined as ≥GG3

Predictor	Measure	HR (95% CI)
Age at diagnosis	Per decade	1.74 (1.41, 2.15)
PSA	Per doubling	1.76 (1.27, 2.47)
Change in PSA	75th vs 25th percentile	1.41 (1.12, 1.71)
Prostate volume	Per doubling	0.41 (0.29, 0.58)
Positive lesion on MRI	Yes vs. No	1.39 (1.18, 1.69)
No. of previous negative Bx	Per additional negative biopsy	0.84 (0.77, 0.91)

PSA = prostate-specific antigen; MRI = magnetic resonance imaging; Bx = prostate biopsy

eTable 5: Time-dependent AUC with the outcome of the model defined as ≥GG3

	Development
**Year**	**PRIAS**
1.5	0.84 (0.79,0.88)
2	0.85 (0.81, 0.89)
3	0.86 (0.83, 0.90)
4	0.87 (0.84, 0.90)
5	0.89 (0.86, 0.92)

© 2025 de Vos II et al. *JAMA Network Open*.

eTable 6: Diagnostic accuracy and net benefit in external validation cohorts when applying the model at moments at which a biopsy was taken (in those cohorts with at least 50 biopsies and more than 10 reclassifications)

Center	Threshold probability	Sensitivity	Specificity	Negative predictive value	Positive predictive value	No. of biopsies delayed (per 1,000 biopsies)	No. of detected reclassifications delayed (per 1,000 biopsies)
**When applied at yr 1.5**							
UCSF	7.5%	94%	29%	80%	62	163	33
UCSF	10%	94%	37%	83%	65	196	33
UCSF	15%	88%	54%	79%	70	304	65
UCSF	20%	75%	66%	68%	73	435	141
JHU	7.5%	97%	32%	99%	10	298	2
JHU	10%	91%	42%	98%	11	393	7
JHU	15%	68%	60%	96%	12	581	24
JHU	20%	47%	71%	94%	12	700	39
Milan	7.5%	89%	35%	94%	20	312	17
Milan	10%	85%	51%	95%	24	452	23
Milan	15%	78%	67%	94%	31	601	35
Milan	20%	70%	78%	93%	38	706	47
MUSIC	7.5%	100%	4%	100%	25	32	0
MUSIC	10%	93%	9%	82%	25	88	16
MUSIC	15%	90%	28%	90%	28	240	24
MUSIC	20%	70%	48%	84%	30	440	72
Sydney	7.5%	100%	48%	100%	38	362	0
Sydney	10%	93%	59%	96%	42	466	17
Sydney	15%	86%	73%	94%	50	586	34
Sydney	20%	57%	75%	85%	42	672	103
**When applied at yr 4**							
UCSF	7.5%	100%	5%	100%	30	39	0
UCSF	10%	100%	11%	100%	31	78	0
UCSF	15%	100%	22%	100%	34	156	0
UCSF	20%	82%	29%	80%	32	260	52
JHU	7.5%	87%	36%	95%	18	331	18
JHU	10%	87%	44%	96%	20	400	18
JHU	15%	74%	60%	94%	23	552	36
JHU	20%	62%	69%	92%	25	651	54
Milan	7.5%	81%	32%	95%	10	309	17
Milan	10%	81%	47%	96%	13	442	17
Milan	15%	81%	59%	97%	16	552	17
Milan	20%	69%	65%	96%	16	624	28
Sydney	7.5%	92%	5%	67%	25	59	20
Sydney	10%	92%	13%	83%	27	118	20
Sydney	15%	92%	42%	94%	35	333	20
Sydney	20%	69%	50%	83%	32	451	78

© 2025 de Vos II et al. *JAMA Network Open*.

eTable 7: Proposed utilisation of the dynamic risk model to enable personalized, risk-based surveillance

**Year***	**1**					**2**				**3**		**4**		**5**		**6**		**7**	
**Month**	0*	3	6	9	12	15	18	21	24	30	36	42	48	54	60	66	72	78	84
**PSA-test**	X	X	X	X	X	X	X	X	X	X	X	X	X	X	X	X	X	X	X
**DRE**	X		X		X		X		X	X	X	X	X	X	X	X	X	X	X
**MRI****	X				X				X		X		X		X		X		X
**Risk assessment with dynamic risk model*****	X				X				X		X		X		X		X		X

* Time of diagnosis

** If PSA-doubling time <10 years: An MRI is recommended every year (only in the years no standard risk assessment is taken). This triggers an additional risk assessment using the dynamic risk model which should be used to decide whether to perform a repeat biopsy

*** Based on the outcome of the risk assessment according to the dynamic risk model an advice will be given on whether or not to take a prostate biopsy. Proposed risk thresholds are:

- <7.5% - advice not to take a repeat biopsy
- 7.5-14.99% - biopsy should be considered, depending on the co-morbidity and patient preference
- ≥15% - advice to perform a repeat biopsy

eFigure 1: Example of calculation of instantaneous change in PSA: First, a linear mixed model is fitted using natural splines for time to allow for a flexible association between time since the start of AS and PSA (depicted by the black line in the graph). The change in PSA is then defined as the rate of change in the predicted PSA level at a certain time-point (for example, the dotted line indicates the change in PSA at 15 years), rather than the average change in PSA, which has historically been used in PSA kinetics. This more refined analysis can more readily reflect sudden increases in PSA, as PSA values measured at later time points have a larger influence on the instantaneous change in PSA compared to PSA values measured earlier. The instantaneous change in PSA can be calculated by taking the first derivative of the fitted natural splines in the regression model.

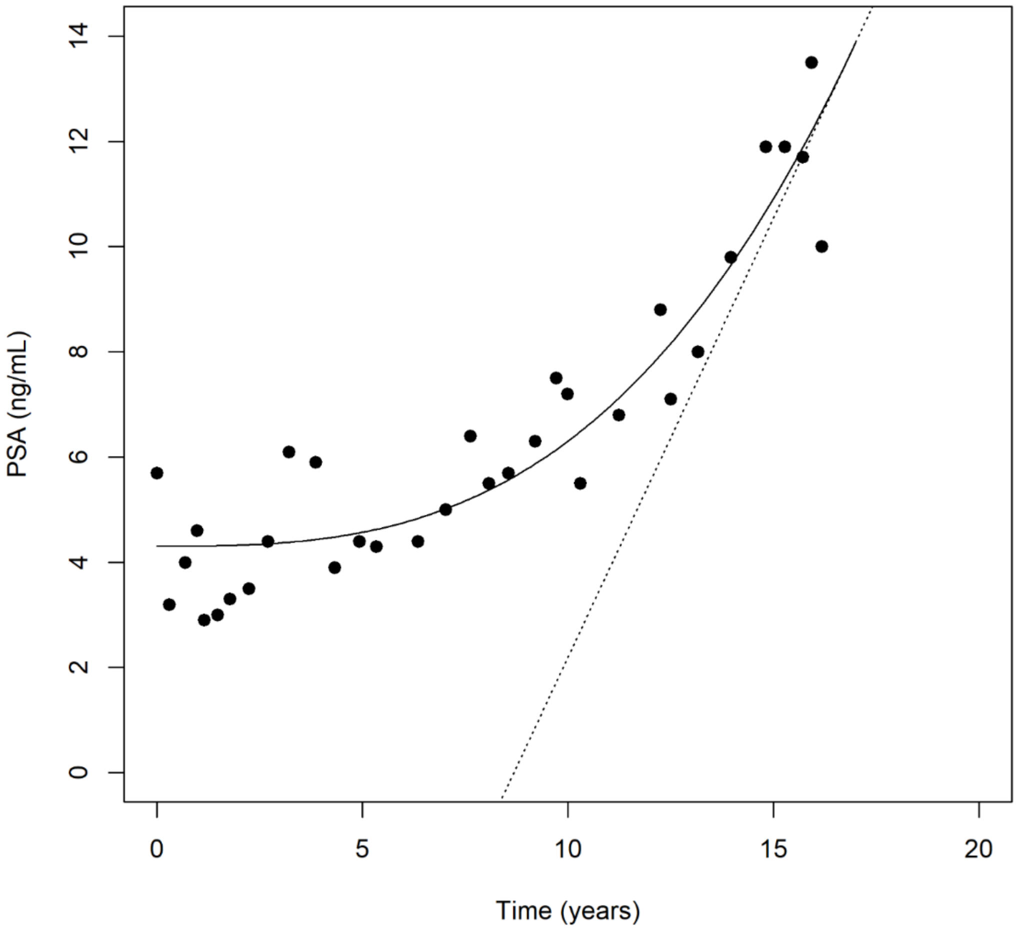

© 2025 de Vos II et al. *JAMA Network Open*.

eFigure 2: Calibration plots comparing predicted versus observed rates of reclassification in different cohorts. Dashes red line is the 95% confidence interval around the observed event rates.
PRIAS = Prostate cancer Research International: Active Surveillance; UCSF = University of California San Francisco; JHU = Johns Hopkins University; MSKCC = Memorial Sloan Kettering Cancer Centre; UCL = University College London; MUSIC = Michigan Urological Surgery Improvement Collaborative

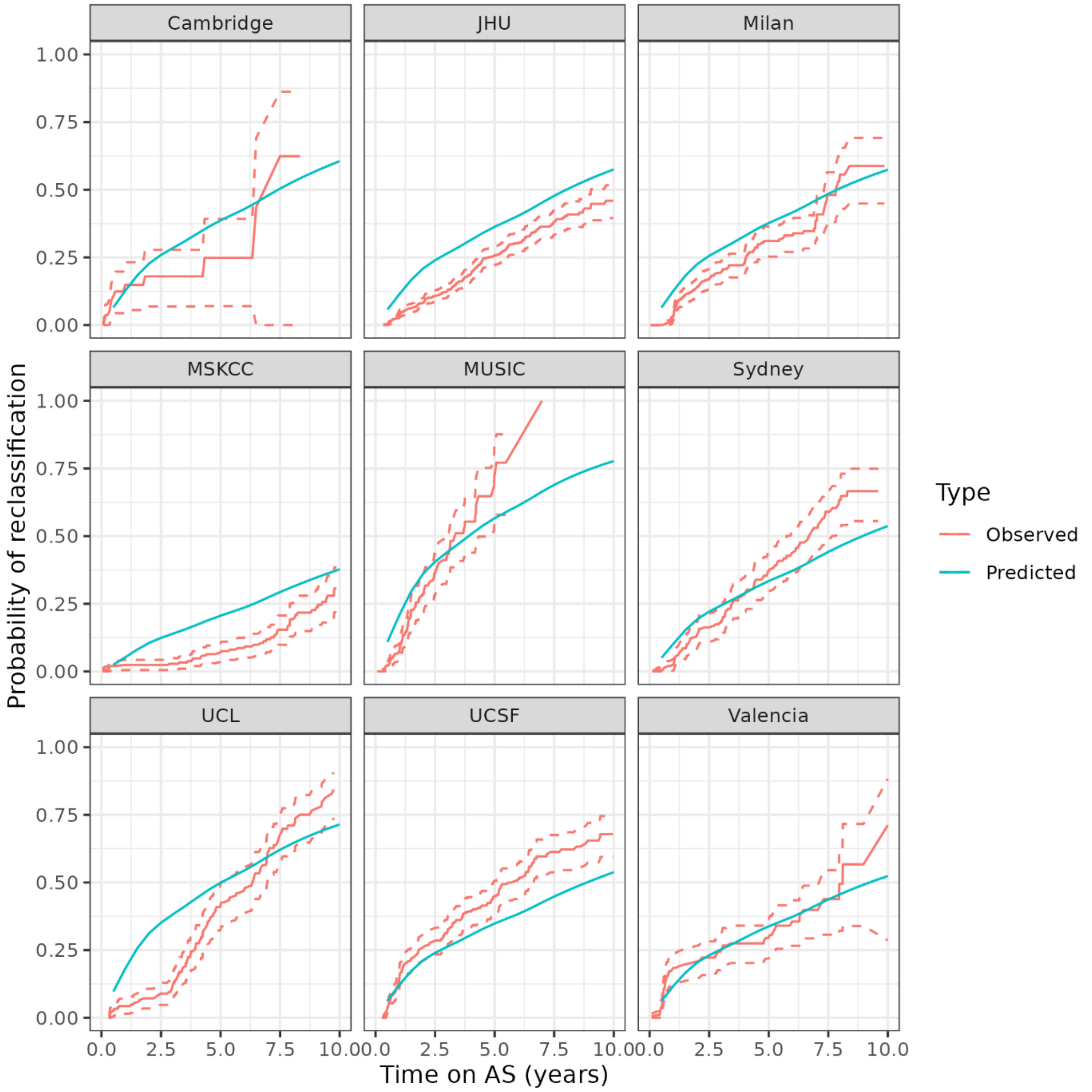

© 2025 de Vos II et al. *JAMA Network Open*.

eFigure 3: Two real-life examples of patients from the PRIAS cohort with PSA progression, but with different results on MRI and a different prostate volume, highlighting the significance of the model's dynamic capability.

Patient A was diagnosed with GG1 disease with a PSA of 5.0 ng/ml, PSA-density of 0.125 ng/ml2, and a suspicious lesion on MRI. The confirmatory biopsy after one year confirmed GG1 disease. Subsequent biopsies at 3 years post-diagnosis, prompted by a PSA rise to 9.9 ng/ml, again identified GG1 on targeted biopsies. However, as the PSA continued to rise to 15 ng/ml, repeat biopsies were conducted at 4 years after diagnosis, revealing reclassification to GG3 which led patient to switch to definitive treatment. Applying the model at the 4-year mark would have indicated a 20% probability of reclassification, which could have strengthened the decision to repeat the biopsy.

In contrast, Patient B, diagnosed with GG1 disease and PSA of 6.0 ng/ml and PSA-density of 0.07 ng/ml2, had no suspicious lesion on MRI at diagnosis. One year after diagnosis, the protocoled confirmatory biopsy showed benign prostate tissue. Similar to Patient A, the PSA rose to 10 ng/ml. However, the prostate volume also increased, resulting in a steady PSA-density of 0.08 ng/ml2. The model would have predicted an 8% risk in reclassification at the four year mark, offering valuable information for considering the possibility to avoid or postpone the repeat biopsy. Ten years after starting AS, biopsies were conducted at a PSA of 15 ng/ml demonstrated no reclassification and the patient is still on active surveillance.

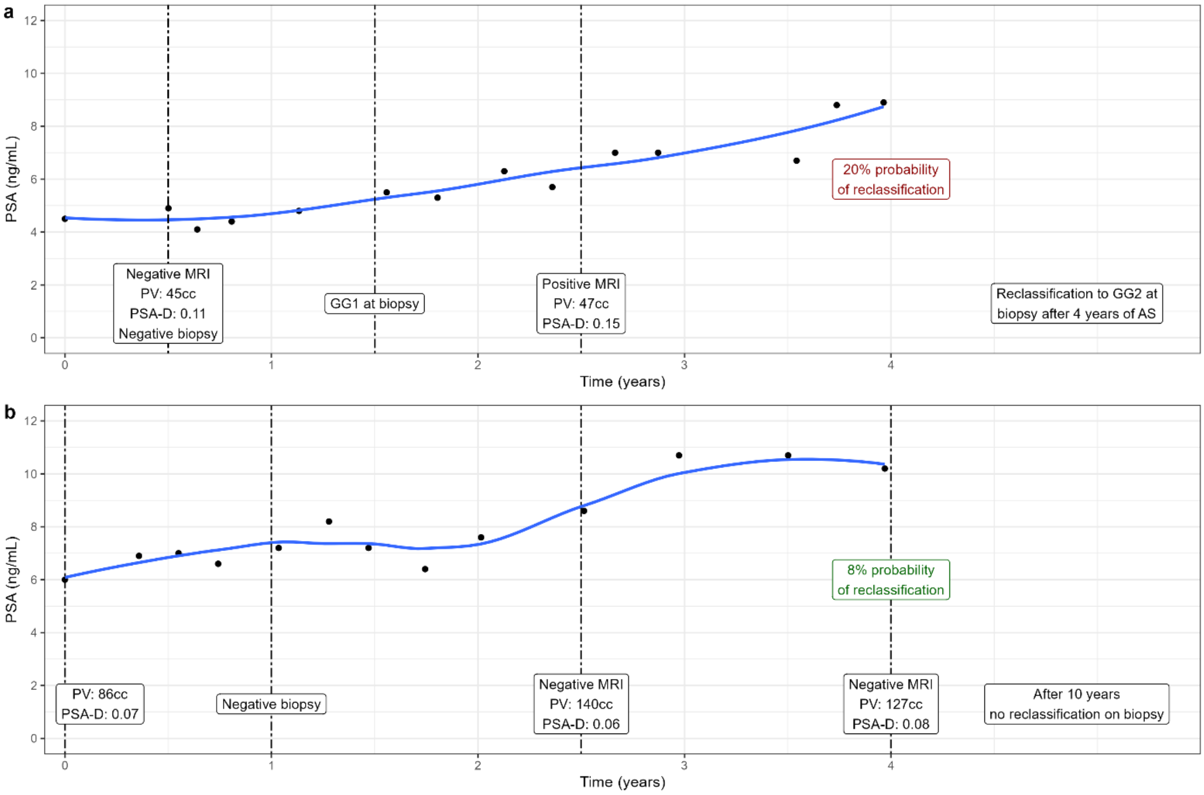

© 2025 de Vos II et al. *JAMA Network Open*.
