# Supplementary material for: Personalized Dynamic Prediction Model for Biopsy Timing in Patients With Prostate Cancer During Active Surveillance
Source: JAMA Netw Open. 2025 Jan 16;8(1):e2454366. doi: 10.1001/jamanetworkopen.2024.54366 (PMC11739991; doi:10.1001/jamanetworkopen.2024.54366)
Supplement: Supplement 2. — Data Sharing Statement [file jamanetwopen-e2454366-s002.pdf]

Data Sharing Statement

de Vos. Personalized Dynamic Prediction Model for Biopsy Timing in Patients With Prostate Cancer During Active Surveillance. *JAMA Netw Open*. Published January 10, 2025. doi:10.1001/jamanetworkopen.2024.54366

Data
Data available: No

Additional Information
Explanation for why data not available: Researchers are encouraged to contact the authors if they are in need of assistance to examine the presented statistical prediction model.
